# Deviations from normative brain white and gray matter structure are associated with psychopathology in youth

**DOI:** 10.1016/j.dcn.2022.101173

**Published:** 2022-11-01

**Authors:** Rikka Kjelkenes, Thomas Wolfers, Dag Alnæs, Linn B. Norbom, Irene Voldsbekk, Madelene Holm, Andreas Dahl, Pierre Berthet, Christian K. Tamnes, Andre F. Marquand, Lars T. Westlye

**Affiliations:** a Department of Psychology, University of Oslo, Norway; b Norwegian Centre for Mental Disorders Research (NORMENT), Division of Mental Health and Addiction, University of Oslo, & Oslo University Hospital, Oslo, Norway; c Oslo New University College, Oslo, Norway; d PROMENTA Research Center, Department of Psychology, University of Oslo, Norway; e Department of Psychiatric Research, Diakonhjemmet Hospital, Oslo, Norway; f Donders Centre for Cognitive Neuroimaging, Donders Institute for Brain, Cognition and Behaviour, Radboud University, Nijmegen, the Netherlands; g Department of Cognitive Neuroscience, Radboud University Medical Centre, Nijmegen, the Netherlands; h Department of Neuroimaging, Centre for Neuroimaging Sciences, Institute of Psychiatry, King’s College London, London, UK; i KG Jebsen Centre for Neurodevelopmental Disorders, University of Oslo, Norway

**Keywords:** Normative modeling, Brain imaging, Adolescence, Cognition, Psychopathology, Pnc

## Abstract

Combining imaging modalities and metrics that are sensitive to various aspects of brain structure and maturation may help identify individuals that show deviations in relation to same-aged peers, and thus benefit early-risk-assessment for mental disorders. We used one timepoint multimodal brain imaging, cognitive, and questionnaire data from 1280 eight- to twenty-one-year-olds from the Philadelphia Neurodevelopmental Cohort. We estimated age-related gray and white matter properties and estimated individual deviation scores using normative modeling. Next, we tested for associations between the estimated deviation scores, and with psychopathology domain scores and cognition. More negative deviations in DTI-based fractional anisotropy (FA) and the first principal eigenvalue of the diffusion tensor (L1) were associated with higher scores on psychosis positive and prodromal symptoms and general psychopathology. A more negative deviation in cortical thickness (CT) was associated with a higher general psychopathology score. Negative deviations in global FA, surface area, L1 and CT were also associated with poorer cognitive performance. No robust associations were found between the deviation scores based on CT and DTI. The low correlations between the different multimodal magnetic resonance imaging-based deviation scores suggest that psychopathological burden in adolescence can be mapped onto partly distinct neurobiological features.

## 1 Introduction

Adolescence confers extensive brain maturation and reorganization across both brain tissue types and regions (Arain et al., 2013, Blakemore, 2012, Norbom et al., 2021). During this period, there is also a sharp increase in the incidence rates of several mental disorders, including schizophrenia and bipolar disorder (Dalsgaard et al., 2020). While the etiologies of common mental disorders are highly complex and largely unknown, accumulating behavioral, neuroimaging and genetic evidence supports a neurodevelopmental contribution (Birnbaum and Weinberger, 2017, Insel, 2010, Kessler et al., 2007). To understand the determinants of both normative and diverging developmental trajectories, more knowledge about the factors that facilitate and impede brain maturation and plasticity, and how these different factors interact during childhood and adolescence is needed.

Cortical thickness and cortical surface area capture various aspects of the coordinated maturational processes. Longitudinal structural magnetic resonance imaging (sMRI) studies have shown that cortical thickness increases during the first years of life, followed by monotonic thinning in most cortical regions from childhood to adulthood (Lyall et al., 2015, Vidal-Pineiro et al., 2020). Cortical surface area expands largely during the two first years, followed by small increases until peaking in late childhood or early adolescence (Gilmore et al., 2018, Tamnes et al., 2017).

While sMRI offers excellent opportunities to study macrostructural and gross morphological brain development, diffusion tensor imaging (DTI) offers a complementary view by providing sensitive measures of the diffusivity and directional coherence of brain tissue. DTI has been used to infer various aspects of gray and white matter microstructure, connectivity, and coherence (Ehrlich et al., 2014, le Bihan, 2003). Developmental studies have found distinct age-related increases in fractional anisotropy (FA) and decrease in mean diffusion (MD) during childhood, adolescence, and early adulthood (Beck et al., 2021, Klingberg et al., 1999, Kwon et al., 2020, Miller et al., 2012, Tamnes et al., 2010, Westlye et al., 2010). These age-related changes have been reported in specific areas of the brain, but robust associations have also been found with global DTI measures (Koenis et al., 2015, Roalf et al., 2020, Tamnes et al., 2011).

The maturation of the brain, captured through DTI and sMRI, reflect various simultaneously interacting microstructural and biological processes, including synaptic pruning and cortical myelination in youth (Natu et al., 2019, Norbom et al., 2021). Supranormal deviations from these maturational patterns in cortical morphometry, and in white matter microstructural properties, have been found to be associated with lower cognitive performance and psychopathology in youth(Alnæs et al., 2020; Asato et al., 2010; Erus et al., 2014; Kaczkurkin et al., 2019; Mewton et al., 2021; Tung et al., 2021).

Careful assessment of individual differences in brain structure and function is important for achieving a better understanding of both normative and aberrant development during adolescence (Foulkes and Blakemore, 2018). Normative modeling is a statistical method used to estimate and characterize the expected age-related trajectories of different biological measures and other phenotypes (Marquand et al., 2019). Individual deviations can then be evaluated in relation to the estimated normative range, offering an individualized quantification of deviation from the expected trajectory.

Previous work has used age and sex based normative models estimated for cortical thickness and brain volume to parse the heterogeneity in brain gray matter in patients with schizophrenia, bipolar disorder, autism spectrum disorder and attention-deficit hyperactivity disorder (Bethlehem et al., 2019, Marquand et al., 2019, Tunc et al., 2019, Wolfers et al., 2020, Wolfers et al., 2018, Zabihi et al., 2019). Normative modeling has also revealed associations between white matter DTI and both preterm birth (Dimitrova et al., 2020) and schizophrenia (Lv et al., 2021), and between cognitive performance and polygenic scores and clinical symptoms of psychopathology in youth (Kjelkenes et al., 2022). Whereas normative modeling has been applied to various data types independently, considering different sources of information or imaging modalities in the same individuals is less common (Fraza et al., 2021). Combining modalities that are sensitive to various aspects of brain maturation has the potential to provide a more extensive characterization of individual brain maturation, which can further be used as a tool for early-risk assessment.

To this end, we used normative modeling and compared individual deviation from estimated age-related norms of features derived from DTI metrics, cortical thickness, surface area and cognitive performance in 1280 participants aged 8–22 years in the Philadelphia Neurodevelopmental cohort (PNC) (Satterthwaite et al., 2014).

Previous normative modelling approaches in the PNC have used regional measures of brain volume (Parkes et al., 2021), cortical thickness and surface area (Cropley et al., 2021) to test for associations with dimensional psychopathology. Our work adds to this previous work by considering different MRI modalities and features yet focus on global characteristics for transparency and simplicity. First, we tested for associations between deviations in the different modalities and assessed the degree to which the various normative models converged for individual participants. Secondly, we tested the associations between the imaging-derived deviation scores and self- reported and collateral caregiver reported symptoms of psychopathology and cognition. Adopting a Bayesian regression framework, we hypothesized that participants deviating in one modality would be more likely to deviate in the other modalities, and that individuals with higher overall deviation scores, would show higher burden of psychopathology symptoms and, given the previously reported link between cognition and psychopathology (Kjelkenes et al., 2022), poorer cognitive function.

## 2 Materials and methods

### 2.1 Participants

PNC is a publicly available population-based sample, with participants between 8 and 21 years from the greater Philadelphia area (Satterthwaite et al., 2016, Satterthwaite et al., 2014). It includes medical history, clinical and cognitive data, as well as neuroimaging for a subset. Recruitment procedures, sample characteristics, clinical, cognitive, and imaging procedures have previously been reported (Satterthwaite et al., 2016, Satterthwaite et al., 2014). Complete cognitive and psychopathology data was available for 6481 participants (3377 girls). We excluded participants with severe medical conditions (*n* = 44, based on rating performed by trained health personnel in the PNC study team), and datasets that did not pass quality assessment leaving a total DTI sample of 1308 participants and a T1-weighted MRI sample of 1400 participants. A total of 1280 participants with all measures available were used to analyze the relationship between deviation scores.

### 2.2 Normative deviation score for cognitive performance

We included a previously estimated cognitive deviation score based on normative modeling (Kjelkenes et al., 2022). This deviation score was calculated through running a principal component analysis on 14 cognitive tests from the PNC computerized test battery assessing general intellectual abilities, executive functioning, episodic memory, complex cognition, social cognition, and sensorimotor speed (Moore et al., 2015) (see Supplemental Fig. S1 for more details). The first component was then extracted as a measure of general cognitive function, which was used to estimate a normative deviation score for cognitive performance (Kjelkenes et al., 2022).

### 2.3 Psychopathology domains

To assess the major domains of psychopathology a computerized, structured interview (GOASSESSS) was used including measures of anxiety, mood, behavioral, eating, and psychosis spectrum disorders. The youths answered themselves, but additional collateral informants were used for participants aged 18 or younger (Calkins et al., 2015). The 129 items were decomposed through an independent component analysis (ICA) using Icasso (Himberg et al., 2004)(see Alnæs et al., 2018 for further description of the procedure). Based on previous work (Alnæs et al., 2018) we included seven independent components (IC) and a proxy for general psychopathology derived from the mean weights across the components. The included ICs included attention problems (IC1), anxiety (IC2), norm-violating behavior (IC3), positive and prodromal psychosis symptoms (IC4), depression, suicide, and psychosis negative symptoms (IC5), mania (IC6), and obsessive-compulsive symptoms (IC7) (Alnæs et al., 2018).

### 2.4 MRI acquisition and analysis

A 3 T Siemens TIM Trio scanner (Siemens Medical Solutions) was used for acquiring the MRI scans (Satterthwaite et al., 2014). Diffusion MRI scans were acquired using a twice-refocused spin-echo single-shot echo-planar imaging sequence (field of view, 240 × 240 mm; matrix, 128 × 128 × 70; 64 diffusion-weighted directions; b = 1000 s/mm^2^; voxel resolution, 1.875 × 1.875 × 2 mm) (Satterthwaite et al., 2014). T1-weighted MRI were collected using a magnetization prepared rapid-acquisition gradient-echo (MPRAGE) sequence (TR=1810 ms; TE=3.51 ms; FoV=180 ×240 mm; Resolution=0.94 ×0.94 ×1.0 mm) (Satterthwaite et al., 2014).

Volumetric segmentation, cortical surface reconstruction and estimation of vertex-wise, and mean cortical thickness as well as total surface area was performed on the T1-weighted images using FreeSurfer (FS) 5.3 (http://surfer.nmr.mgh.harvard.edu) (Fischl, 2012). The quality of the cortical reconstructions was assessed using a flagging procedure that was based on Euler number (Rosen et al., 2018) and a robust principal component analysis for detecting signal-to-noise and segmentation outliers (Hubert and Engelen, 2004). We carefully inspected the flagged datasets and performed minor edits as appropriate. We excluded scans from 63 participants due to poor image quality. Klikk eller trykk her for å skrive inn tekst. We processed the diffusion-weighted imaging using tools from FMRIB Software Library´s (FSL´s) Diffusion Toolbox (Jenkinson et al., 2012) including corrections for susceptibility induced distortions, head movements and eddy current induced distortions using topup (http://fsl.fmrib.ox.ac.uk/fsl/fslwiki/topup) and eddy (http://fsl.fmrib.ox.ac.uk/fsl/fslwiki/eddy) (Andersson and Sotiropoulos, 2016), Next, using dtifit in FSL, FA, eigenvector and eigenvalue maps were computed by fitting a tensor model to the corrected diffusion data and used to derive maps for axial (L1, the principal diffusion tensor imaging eigen value), mean and radial diffusivity. We then implemented tract based spatial statistics (TBSS, (Smith et al., 2006)) to transform all maps to the FMRIB58_FA template and skeletonize the maps. TBSS processing was also applied to the non-FA DTI measures (L1, MD, RD) and we computed the mean of these measures across the whole skeleton using FSL tools. Data quality was assessed by eyeballing snapshots of individual FA maps, and flagging the images with obvious problems for further evaluation. After review 91 participants were excluded.

### 2.5 Normative modeling

We estimated the normative models using python version 3.8 (https://www.python.org/), and the pcntoolkit version 0.21 (Rutherford et al., 2022). We used the Bayesian linear regression (BLR) algorithm within the pcntoolkit (Fraza et al., 2021, Huertas et al., 2017) where we aimed to predict each brain-derived measures using age and sex as covariates and applied common B-spline basis expansion to the age variable with cubic splines with three evenly spaced knot points. We used the Powell method for optimization. All selected features showed evenly distributed variations across the age range and standard Gaussian warping was thus applied (see S2 for distribution plots). Performance of the normative model was estimated out of sample using 10-fold cross validation. The deviations from each normative model were quantified by computing a Z-score reflecting the difference between the predicted and the observed feature normalized by the uncertainty (Fraza et al., 2021, Huertas et al., 2017).

**Fig. 1:**
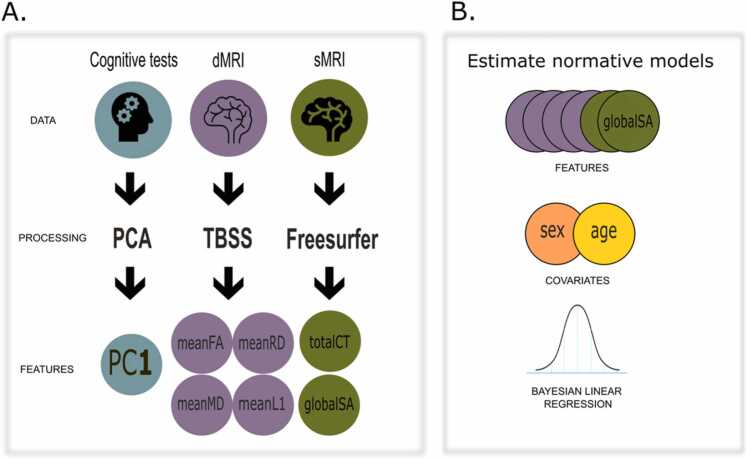
A) Overview of the different raw data included, the processing steps employed, and the final features added to the analysis. B) Overview of the estimation of the normative models. dMRI = diffusion magnetic resonance imaging, sMRI = structural magnetic resonance imaging, PCA = principal component analysis, TBSS = tract-based spatial statistics, FA = fractional anisotropy, MD = mean diffusivity, RD = radial diffusivity, L1 = the principal diffusion tensor imaging eigen value, CT = cortical thickness, SA = surface area.

For cortical morphometry data we estimated normative models for mean cortical thickness, and total cortical surface area. Here the BLR predicted 21.5 % of the variance of the observed mean thickness score out-of-sample, and 29.3 % for surface area. For DTI we estimated normative models using FA, MD, RD, and L1 averaged across the TBSS skeleton. Here the BLR predicted 21.8 %, 36.0 %, 33.6 %, and 23.8 % of the variance of the respective modality out-of-sample. Additional performance measures are provided in the Supplementary materials (table S3). (Fig. 1).

### 2.6 Statistical analysis

We calculated Pearson correlations between the different deviation scores to quantify their pair-wise associations. We applied a Bayesian approach using the brms (Bürkner, 2018, Bürkner, 2017) package in R (R Core Team, 2020) to examine linear associations between the deviation scores derived from the different modalities, the psychopathology scores, and the cognitive deviation score. The deviation scores from each of the normative trajectories (i.e., subject-level Z-statistics) were included as the dependent variable and age, sex and the clinical domain scores were entered as independent variables. Prior to analysis the residuals of the psychopathology domain scores and age were standardized, by subtracting their mean and dividing by their standard deviation. The Savage-Dickey density ratio method was used to calculate the Bayes Factors (BF) (Wagenmakers et al., 2010), which indicates the strength of evidence in favor of the null hypothesis or the alternative hypothesis. BF= 1 can be interpreted as evidence in either direction. The following values can be interpreted as weight towards the alternative hypothesis with the following strengths: 0.3–1 (anecdotal), 0.1–0.3 (moderate), 0.03–0.1 (strong), 0.01–0.03 (very strong), < 0.01 (extreme). BF> 1 provides evidence towards the null hypothesis: 1–3 (anecdotal), 3–10 (moderate), 10–30 (strong), 30–100 (very strong), > 100 (extreme) (Wagenmakers et al., 2010).

## 3 Results

### 3.1 Associations between deviation scores from different modalities

**Fig. 2:**
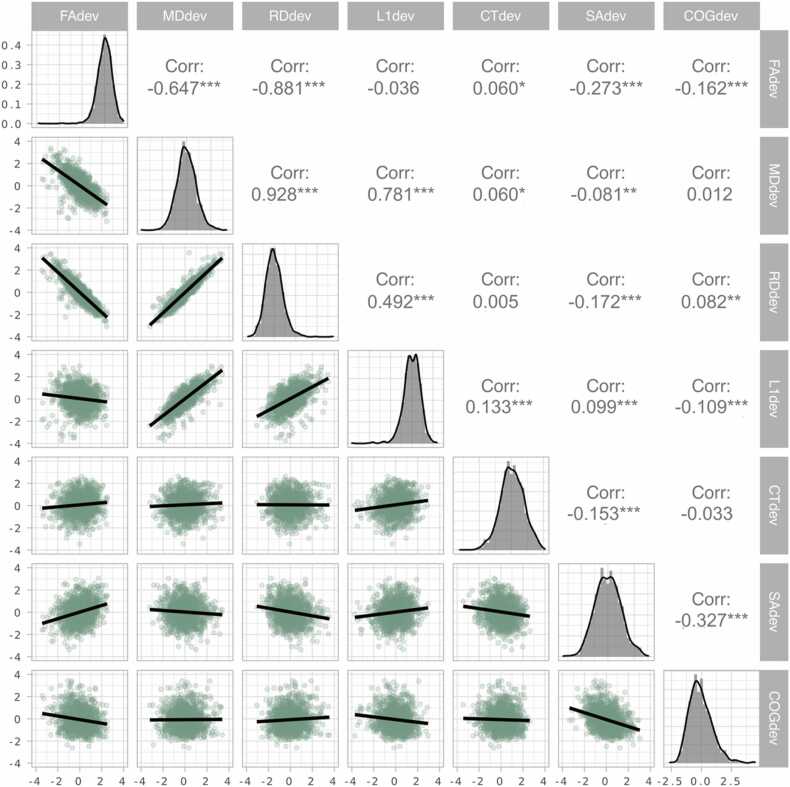
Pairwise scatter plots. In the diagonal section we see density plots for the deviation scores from the different modalities. The scatter plots show the pairwise relationships between the deviation scores. The corresponding Pearson correlations are listed in the upper diagonal. FA_dev_= mean fractional anisotropy deviation score, L1_dev_ = mean axial diffusivity deviation score, MD_dev_ = mean mean diffusivity deviation score, RD_dev_= mean radial diffusivity deviation score, CT_dev_ = global cortical thickness deviation score, SA_dev_ = global surface area deviation score, COG_dev_ = cognitive deviation score, Corr= correlation, * p < .05, ** = p < 0.01, * ** = p < 0.001.

Fig. 2 shows the pairwise Pearson’s correlations between the different deviation scores, revealing high correlations between the mean FA deviation (FA_dev_) score and the DTI derived measures mean MD deviation (MD_dev_) score (r = −.65) and the mean RD deviation (RD_dev_) score (r = −.88). The correlation between FA_dev_ and the mean L1 deviation score (L1_dev_) was low (r = −.03). L1_dev_ had a higher correlation with MD_dev_ (r = .78) and RD_dev_ (r = .49). The analysis revealed low correlations between the cortical thickness deviation (CT_dev_) score and the other deviation scores (r = 0.005–0.13), with the highest correlation with total surface area deviation (SA_dev_). For SA_dev_ the analysis revealed highest correlations with the cognitive deviation score (r = −.32) and FA_dev_ (r = .27).

### 3.2 Associations between normative models of DTI-based white matter structure, cognitive deviations and psychopathology

**Fig. 3:**
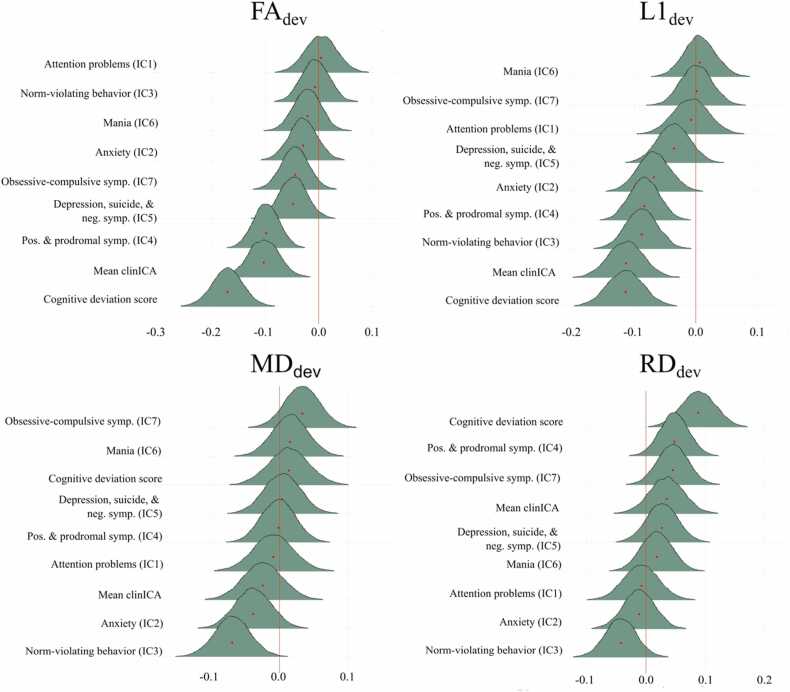
Association between cognitive and psychopathology domain scores and DTI deviation scores, including deviations score from FA_dev_, L1_dev_,MD_dev_, and RD_dev_. FA_dev_= mean fractional ansisotropy deviation score, L1_dev_ = mean axial diffusivity deviation score, MD_dev_ = mean diffusivity deviation score, RD_dev_= mean radial diffusivity deviation score. Mean clinICA=generalized psychopathology, symp=symptoms, Pos= positive, neg= negative.

The posterior distributions for the associations between the psychopathology domain scores, the cognitive deviation score and the deviation scores derived from different DTI-based measures are illustrated in Fig. 3. These results showed extreme evidence of a negative association between cognition and FA_dev_ (BF=<.01, B=−.170), indicating lower cognitive performance with more negative FA deviation. There was also extreme evidence for an association between FA_dev_ and the psychosis positive and prodromal symptoms (BF<0.01, B=−.098), and strong evidence for an association with the general psychopathology factor (BF=0.06, B=−.102). The comparison revealed moderate evidence for no association between the FA_dev_ and depression, suicide, and negative symptoms (BF=4.49, B=−.044), obsessive-compulsive symptoms (BF=4.49, B=−0.044), and anxiety (BF=9.90, B=−0.029). Lastly, the test indicated strong evidence for no association between FA_dev_ and attention problems (BF=16.36, B=0.005), norm-violating behavior (BF=18.60, b=−.006), and mania (BF=14.06, B=−.021).

For L1_dev_, the analysis revealed extreme evidence of an association with general psychopathology (BF<0.01, B=−.113) and cognitive deviation (BF<0.01, B=−.115), strong evidence of an association with psychosis positive and prodromal symptoms (BF=.06, B=−.083) and norm-violating behaviour (BF=.08, B=−.088), and anecdotal evidence of an association with anxiety (BF=0.80, B=−.068). The results also suggested moderate evidence for no association with depression, suicide, and negative symptoms (BF=7.19, B=−.036) and strong evidence for no association with attention problems (BF=16.35, B=.008), obsessive-compulsive symptoms (BF= 19.28, B=−.001), and mania (BF=19.43, B=−.007).

For MD_dev,_ anecdotal evidence was found for an association with norm-violating behaviour (BF=0.62, b=−.068), moderate evidence for no association with anxiety (BF=6.87, B=−.038) and obsessive-compulsive symptoms (BF=8.15, B=.033), and strong evidence for no association with attention problems (BF=16.45, B=−.009), psychosis positive and prodromal symptoms (BF=19.80, B=−.001), depression, suicide, and negative symptoms (BF=18.37, B=.004), mania (BF=15.23, B=.016), general psychopathology (BF=11.53, B=−.102) and cognitive deviation (BF=14.86, B=.014).

For RD_dev_, the tests confirmed moderate evidence of an association with cognitive deviation (BF=0.15, B=.088), moderate evidence for no associations with norm-violating behaviour (BF=5.11, B=−.042), positive and prodromal symptoms (BF=3.10, B=.048), obsessive-compulsive disorder (BF=4.401, B=.045), and general psychopathology (BF= 8.72, B=.035), and strong evidence for no association with attention problems (BF=16.08, B=−.008), anxiety (BF=16.85, B=−.012), depression, suicide, and negative symptoms (BF=10.96, B=.027), and mania (BF=14.99, B=.018).

### 3.3 Associations between normative deviations in cortical morphometry, cognitive deviations and psychopathology

**Fig. 4:**
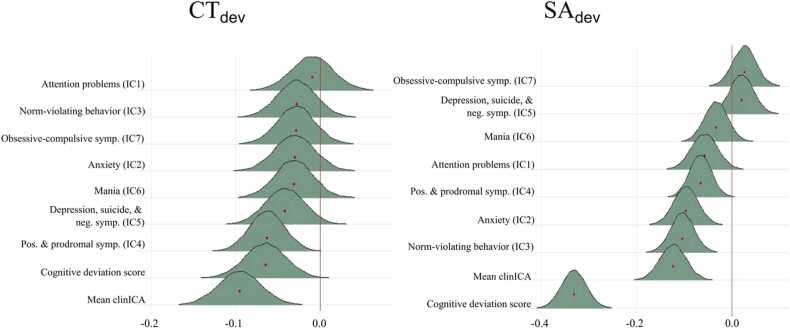
Association between cortical thickness deviation score (left) and surface area deviation score (right) and psychopathology. CT_dev_ = global cortical thickness deviation score, SA_dev_ = global surface area deviation score, Mean clinICA=generalized psychopathology, symp=symptoms, Pos= positive, neg= negative.

Fig. 4 shows the posterior distributions reflecting the associations between deviation scores in cortical morphometrics, cognition, and psychopathology. For CT_dev_, the tests revealed extreme evidence of a negative association with general psychopathology (BF <0.01, B=−.10). This indicates that higher general psychopathology is linked with lower cortical thickness. We found anecdotal evidence for an association with positive and prodromal symptoms (BF=0.71, B=−.062) and with the cognitive deviation score (BF=.97, B=−.068). In addition we found strong evidence for no association with attention problems (BF=16.71, B=−.012) and anxiety (BF=10.50, B=−.028). The test provided moderate evidence for no association with norm-violating behaviour (BF=7.60, B=−.034), obsessive-compulsive (BF=9.50, B=−.031), and mania (BF=8.94, B=−.032). Finally, we found anecdotal evidence for no association with depression and suicide and negative symptoms (BF=2.69, B=−.050).

For SA_dev_, the tests revealed extreme evidence of an association with anxiety (BF<.001, B=−.097), norm-violating behaviour (BF <0.01, B=−.104), general psychopathology (BF<.001, B=−.124) and cognitive deviation (BF<.001, B=−.33), anecdotal evidence of an association with positive and prodromal symptoms (BF=0.44, B=−.066) was also found. In addition we found strong evidence for no association with depression (BF=17.17, B=.014) and obsessive-compulsive disorder (BF=14.70, B=.021), moderate evidence for no association with mania (BF=8.25, B=−.033), and anecdotal evidence of an association with attention problems (BF=2.34, B=−.054).

## 4 Discussion

The emergence of mental disorders during adolescence and young adulthood is likely preceded and partly mediated by deviations from typical brain maturation. We used structural brain imaging and normative modeling to examine relationships between individual deviations from expected brain white and gray matter trajectories derived from different MRI modalities, cognitive function, and mental healthy symptoms spanning various domains.

Our analyses revealed low correlations between normative deviation scores derived from DTI-based white matter structure and cortical morphometrics. Overall, this supports a multidimensional view on brain maturation during childhood and adolescence indicating that different driving factors of brain development have different impact on gray and white matter maturation, respectively, serving as a reminder that individuals deviating in one modality are not necessarily deviating in another. Next, while all MRI based deviation scores were associated with a questionnaire-derived proxy of generalized psychopathology, the symptom measures reflecting specific domains of psychopathology showed differential associations with the imaging-based deviation scores. This confirms that individual departures from typical brain developmental trajectories are related to symptoms of psychopathology and demonstrates that different clinical domains and symptoms might map onto different imaging modalities and brain tissue types. Lastly, we observed significant associations between all brain derived deviation scores and the cognitive deviation score, with the strongest association for surface area. This supports the sensitivity of surface area as a proxy for global maturational processes supporting neurocognitive development, and the close link between cognition and brain maturation.

### 4.1 Normative deviations across different modalities show little overlap

Our analysis revealed overall evidence of low to no correlations between deviation in cortical thickness and deviations in the other imaging modalities. Thus, our findings partly diverge from previous reports of weak to moderate correlations between mean cortical thickness and both mean FA and mean MD among healthy participants aged 8–30 years (Tamnes et al., 2011). The lack of correlations between modalities also contrasts the general assumption that cortical gray and white matter developmental processes reflect coordinated and closely intertwined biological mechanisms with similar sensitivity to cognitive development and psychopathology (Jeon et al., 2015).

Our analyses revealed a negative correlation between the deviation scores in cortical thickness and surface area. Developmental changes in cortical thickness might partly be a consequence of the underlying white matter tissue growth e.g., due to myelination, and subsequent stretching of the cortex, making it thinner, and at the same time expanding the surface area (Seldon, 2005). This balloon model would explain the observed negative relationship between CT_dev_ and SA_dev_. Low correlations between surface area and cortical thickness have been reported during the first two years of life (Lyall et al., 2015), less consistent and more regionally specific associations during childhood and adolescence (Tamnes et al., 2017), and largely negative correlations across adulthood (Hogstrom et al., 2013). Combined, these studies suggest that both the strength and direction of the association might change with age (Norbom et al., 2021). Considering this balloon model, apparent cortical thickness likely reflects a combination of the biology of the cortical gray matter and the dynamics and mechanical forces of the underlying white matter stretching the cortical mantle. It has been suggested that such “cortical stretching” reflects a phylogenetic principle of the benefits of maximizing surface area and gyrification rather than increase thickness to facilitate brain connectivity and development (Hogstrom et al., 2013). While the lack of associations between DTI-based and cortical morphometry normative deviations does not support a simple link between white matter architecture and cortical development, previous studies have shown weak correlations between white matter volume and FA, indicating these indices are sensitive to different characteristics of white matter integrity and growth (Fjell et al., 2008).

The interpretations of shared variance, or a lack thereof, between imaging modalities, should be done with caution. The signal captured by each MRI modality is likely to be sensitive to partly independent and partly non-independent genetic and biological processes (Rakic, 1988, Smith et al., 2021). The correspondence between different MRI based measures are also likely to be influenced by developmental, aging-related, and pathological processes, which may or may not act globally. In line with the current observations, a prior study reported a positive correlation between global cortical thickness and global FA among patients diagnosed with schizophrenia, but not among healthy controls (Sasamoto et al., 2014). This may indicate that high correspondence between imaging modalities primarily reflects disease-specific and possibly global pathological processes that are not present in the current young, undiagnosed sample. It could also mean that some of the variance shared between modalities is due to global age-related effects. Data fusion approaches often identify global age-related effects across modalities (Groves et al., 2012, Llera et al., 2019), which are effectively accounted for using age and sex based normative modeling. Another possible explanation is that the associations are regionally specific. This is supported by previously reported regional fusing between modalities in a lifespan sample (Groves et al., 2012), regional associations between cortical thickness and FA in healthy adults (Ehrlich et al., 2014), and correlations between cortical thickness and FA in the frontal lobe in youth (Jeon et al., 2015).

While the current associations between modalities were weak, there is wide support for the benefits of combining multiple MRI modalities when studying brain development, aging, and disease (Fernandez-Cabello et al., 2022, Groves et al., 2012, Rokicki et al., 2021, Smith et al., 2020). Combining measures increases sensitivity to age and improves discrimination accuracy for common disorders of the brain (Doan et al., 2017, Rokicki et al., 2021) and has been shown to boost gene discovery for brain imaging features (van der Meer et al., 2020),. Our findings support that adolescent brain development as captured using cortical morphometry and DTI-based white matter structure comprises several multidimensional and heterogeneous processes. This corroborates previous work demonstrating the importance of studying multiple brain and behavioral phenotypes when characterizing individuals in relation to normative developmental trajectories.

### 4.2 Associations between brain deviations and general and domain-specific psychopathology

The second aim of our analysis was to examine associations between brain deviation scores and both generalized and specific domains of psychopathology. We found evidence for a negative association between the deviation scores derived from cortical thickness, surface area, FA and L1 and a questionnaire-derived generalized psychopathology measure. This suggests that broad dimensions of psychopathology cutting across diagnostic boundaries are associated with deviations in cortical morphometry and white matter properties. This is in line with prior population-based studies showing relationships between generalized psychopathology and brain volume and surface area in both children aged 9–10 years (Mewton et al., 2021) and in the PNC sample (Kaczkurkin et al., 2019, Parkes et al., 2021). DTI associations with psychopathology have also been reported in both youth from the PNC sample (Alnæs et al., 2018) and in children aged 6–10 years (Neumann et al., 2020).

In addition to generalized psychopathology our analyses also revealed associations between specific domains of psychopathology and brain deviations. Strongest evidence was found for an association between psychosis positive and prodromal symptoms and normative deviations in FA, indicating lower mean FA with higher symptom levels. While it is unclear to which degree the clinical domain scores reflect future risk of developing schizophrenia or other severe psychotic disorders, prior studies have reported similar global reductions in FA in both early psychosis and in the prodromal phase in youth aged 11–20 from the PNC sample (Hegarty et al., 2019), and among early-onset (Tamnes and Agartz, 2016) and adult patients with schizophrenia compared to healthy controls (Kelly et al., 2018, Tønnesen et al., 2018).

We also found norm-violating behavior to be associated with L1_dev_ and SA_dev_. This is in line with a study of 7124 children aged 9–11 years from the ABCD study reporting a negative association between externalizing problems and surface area (Fernandez-Cabello et al., 2022). For SA_dev_ we also found evidence for an association with the anxiety domain score. This is in line with a study showing a similar association between anxiety and surface area in youths aged 7–20 years from the PING study (Newman et al., 2016). Here, an additional association between cortical thickness and anxiety was reported, which we did not find evidence for in the current study.

A recent longitudinal study predicted neurodevelopmental trajectories using normative modeling on both global and regional cortical thickness and surface area based on 5454 brain scans from 4415 participants aged 6–16 years (Blok et al., 2022). They reported similar associations between psychopathology and cortical volume, surface area and cortical thickness, and found that these associations appeared rather global. In line with our observations, they reported low correlation between deviations in cortical thickness and surface area, and a larger association between specific psychopathology and surface area than with cortical thickness. Importantly, psychopathology symptoms were overall related to negative deviations for cortical volume and surface area and to positive deviations for cortical thickness, supporting our findings. Thus, the previous and current studies provide converging evidence for the importance of surface area for detecting brain maturational deviations, both in relation to generalized or more specific domains of psychopathology.

### 4.3 Brain normative deviations and cognitive function

Deviation scores derived from most imaging features were found to be associated with the cognitive deviation score, indicating higher cognitive performance with deviations reflecting a more mature brain, with small to medium effect sizes. The exception was cortical thickness, where we found a weak negative association, indicating that having a thicker cortex was linked to better cognitive performance. Prior studies have supported a relationship between cognitive performance and surface area in 10-year-old children (Patel et al., 2022), and white matter DTI in both the PNC sample (Tang et al., 2017) and during childhood and adolescence (Goddings et al., 2021). Studies assessing the link between cognition and cortical thickness have revealed mixed results, with some reporting a positive association (Burgaleta et al., 2013, Choi et al., 2008, Karama et al., 2013, Karama et al., 2011), and others no significant associations (Tamnes et al., 2011). A study including 515 middle-aged twins reported that the overall genetic and phenotypic associations between brain volume and general cognitive ability was primarily accounted for by surface area rather than cortical thickness (Vuoksimaa et al., 2014). Similar to our findings, a study on 9623 kids aged 9–10 years from the ABCD study utilizing canonical correlation analysis (CCA) reported links between higher cognitive ability and brain development across modalities (Modabbernia et al., 2021). Other CCA-studies in youths have also linked cross modality patterns of brain development, cognition, and psychopathology across disorders (Alnæs et al., 2020, Voldsbekk et al., 2022). These studies, and ours, suggest that deviations from expected cognitive development precedes disease onset and therefore should be viewed as a cross-diagnostic risk factor for mental illness.

### 4.4 Limitations

To ease interpretation and reduce analytical complexity we only included global MRI measures. It is possible that including regional measures would have revealed different patterns of associations. Future studies may be able to pursue a regional approach across modalities. Further validation of the models outside of the PNC are needed to be able to generalize the findings to a wider population. Longitudinal data are also needed to validate the normative trajectories and their predictive value for future mental health issues and various real-life outcomes. The normative modeling framework is ideal for estimating age trajectories beyond adolescence, and future studies may benefit from including both a younger and older study population. Various environmental and genetic variables are likely to explain and modulate the associations between brain imaging, cognition, and psychopathology in youth, including sociodemographic variables such as poverty and quality of schooling, perinatal factors such as birth weight or premature birth (Alnæs et al., 2020, Modabbernia et al., 2021), and maturational processes including onset and rate of puberty (Holm et al., 2022). Future studies may be able to model the instantaneous influence of a range of modulating factors to increase the precision, relevance, and predictive value of the output from the normative modeling framework.

## 5 Conclusion

This work shows that the normative modeling framework can be used to capture multimodal and idiosyncratic patterns of deviations from age-expected white and gray matter features during development and highlights the potential for early detection of at-risk youths. Normative deviations from age-expected white and gray matter trajectories were associated with cognitive performance and general psychopathology symptom burden in youth, in addition to positive and prodromal symptoms of psychosis. The weak associations between the different multimodal MRI based deviation scores support the complexity of adolescent brain maturation and shows that one modality cannot capture all associations and suggest that psychopathological burden in adolescence may map onto partly distinct neurobiological features.

## Declaration of Competing Interest

The authors declare that they have no known competing financial interests or personal relationships that could have appeared to influence the work reported in this paper.

## Data Availability

Code will be made available before publication. Access to the PNC data has been provided through: dbGaP Authorized Access (https://dbgap.ncbi.nlm.nih.gov/).
