# Supplementary material for: Deviations from normative brain white and gray matter structure are associated with psychopathology in youth
Source: Dev Cogn Neurosci. 2022 Nov 1;58:101173. doi: 10.1016/j.dcn.2022.101173 (PMC9637865; doi:10.1016/j.dcn.2022.101173)
Supplement: Supplementary file 1 — Supplementary material. [file mmc1.docx]

**Deviations from normative brain white and gray matter structure are associated with psychopathology in youth**

***Supplemental Information***

**Supplemental table S1.** All included cognitive test measures and their contributions to the first five principal components from the Principal Component Analysis.


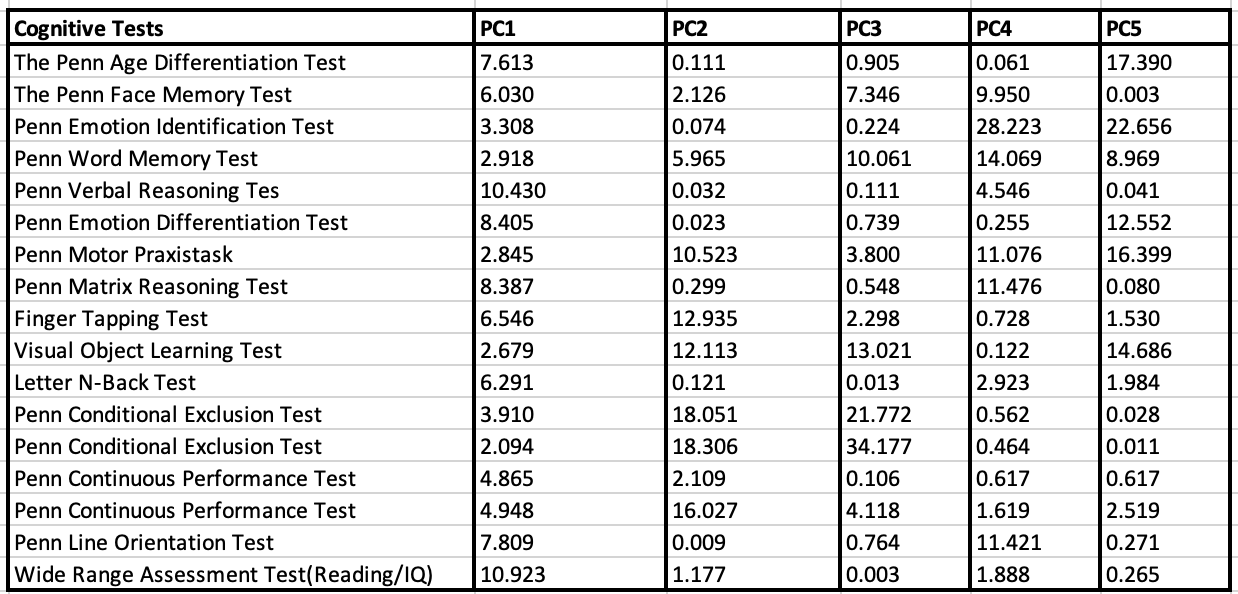


**Supplemental figure S2.** Distributions of the different raw imaging modalities

**
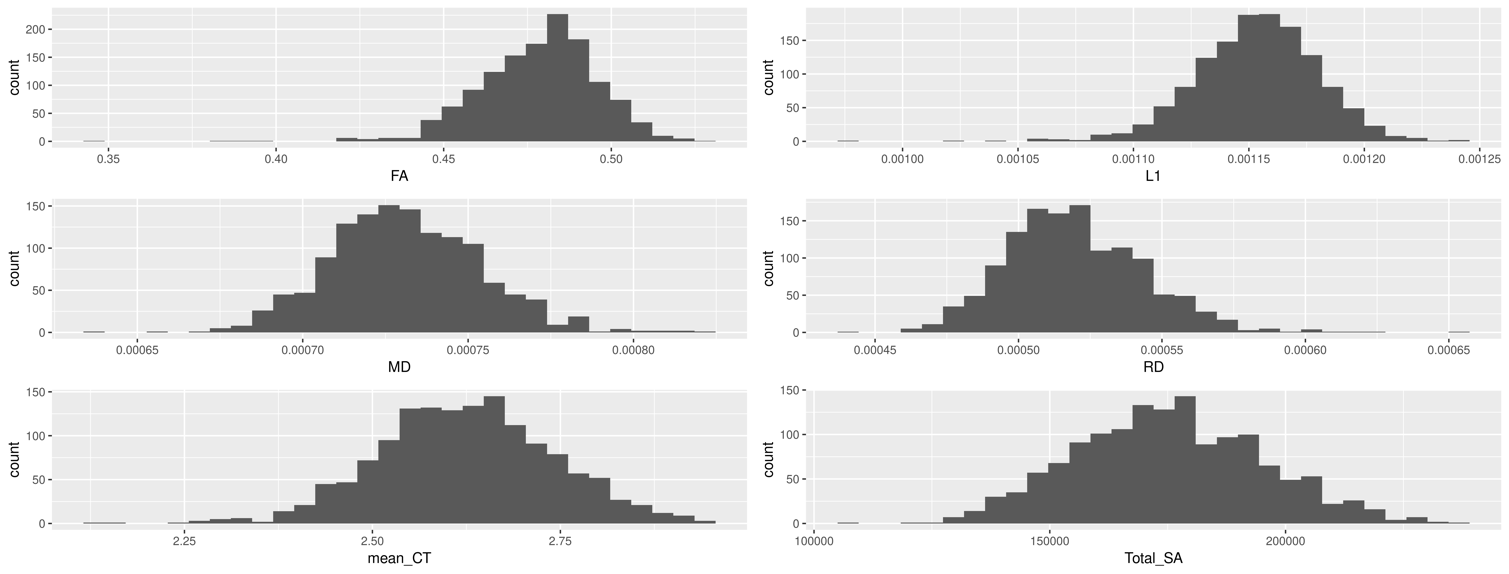
**

**Supplemental table S3.** Performance scores from estimation of the Normative models for the various modalities. EXPV = explained variance, Rho = Pearson correlation between true/predicted responses

, SMSE = standardized mean square error, FA_dev_= mean fractional ansisotrophy deviation score, L1_dev_ = mean axial diffusivity deviation score, MD_dev_ = mean medial diffusivity deviation score, RD_dev_= mean radial diffusivity deviation score.

|  | FA_dev_ | MD_dev_ | RD_dev_ | L1_dev_ | CT_dev_ | SA_dev_ |
| --- | --- | --- | --- | --- | --- | --- |
| EXPV | 0.22 | 0.36 | 0.34 | 0.24 | 0.22 | 0.29 |
| Rho | 0.47 | 0.60 | 0.58 | 0.49 | 0.46 | 0.54 |
| SMSE | 0.78 | 0.64 | 0.66 | 0.76 | 0.78 | 0.71 |

**Supplemental table S4**. Summary stats for Bayesian statistical analysis with the DTI-derived measures

| *y* | *clin* | *estimate* | *lower95* | *upper95* | *p_higher_0* | *p_lower_0* | *evidence_0* | *BF_0* | *loo_elpd_diff* | *loo_se_diff* | *max_rhat* |
| --- | --- | --- | --- | --- | --- | --- | --- | --- | --- | --- | --- |
| z_FA | clinIC1 | 0.00 | -0.05 | 0.06 | 0.56 | 0.44 | 16.36 | 0 | 0 | 0 | 1.00 |
| z_FA | clinIC2 | -0.03 | -0.08 | 0.02 | 0.13 | 0.87 | 9.90 | 0 | 0 | 0 | 1.00 |
| z_FA | clinIC3 | -0.01 | -0.05 | 0.04 | 0.40 | 0.60 | 18.59 | 0 | 0 | 0 | 1.00 |
| z_FA | clinIC4 | -0.10 | -0.14 | -0.05 | 0.00 | 1.00 | 0.00 | 0 | 0 | 0 | 1.00 |
| z_FA | clinIC5 | -0.05 | -0.10 | 0.00 | 0.04 | 0.96 | 3.43 | 0 | 0 | 0 | 1.00 |
| z_FA | clinIC6 | -0.02 | -0.07 | 0.03 | 0.21 | 0.79 | 14.06 | 0 | 0 | 0 | 1.00 |
| z_FA | clinIC7 | -0.04 | -0.09 | 0.01 | 0.04 | 0.96 | 4.49 | 0 | 0 | 0 | 1.00 |
| z_FA | mean_clinICA | -0.10 | -0.16 | -0.05 | 0.00 | 1.00 | 0.06 | 0 | 0 | 0 | 1.00 |
| z_FA | z | -0.17 | -0.23 | -0.11 | 0.00 | 1.00 | -0.00 | 0 | 0 | 0 | 1.00 |
| z_L1 | clinIC1 | -0.01 | -0.07 | 0.05 | 0.40 | 0.60 | 16.35 | 0 | 0 | 0 | 1.00 |
| z_L1 | clinIC2 | -0.07 | -0.12 | -0.02 | 0.01 | 0.99 | 0.80 | 0 | 0 | 0 | 1.00 |
| z_L1 | clinIC3 | -0.09 | -0.14 | -0.04 | 0.00 | 1.00 | 0.08 | 0 | 0 | 0 | 1.00 |
| z_L1 | clinIC4 | -0.08 | -0.13 | -0.03 | 0.00 | 1.00 | 0.06 | 0 | 0 | 0 | 1.00 |
| z_L1 | clinIC5 | -0.04 | -0.09 | 0.02 | 0.09 | 0.91 | 7.18 | 0 | 0 | 0 | 1.00 |
| z_L1 | clinIC6 | 0.01 | -0.04 | 0.06 | 0.60 | 0.40 | 19.43 | 0 | 0 | 0 | 1.00 |
| z_L1 | clinIC7 | 0.00 | -0.05 | 0.05 | 0.51 | 0.49 | 19.25 | 0 | 0 | 0 | 1.00 |
| z_L1 | mean_clinICA | -0.11 | -0.17 | -0.06 | 0.00 | 1.00 | 0.00 | 0 | 0 | 0 | 1.00 |
| z_L1 | z | -0.11 | -0.17 | -0.06 | 0.00 | 1.00 | 0.01 | 0 | 0 | 0 | 1.00 |
| z_RD | clinIC1 | -0.01 | -0.07 | 0.05 | 0.39 | 0.61 | 16.08 | 0 | 0 | 0 | 1.00 |
| z_RD | clinIC2 | -0.01 | -0.06 | 0.04 | 0.33 | 0.67 | 16.85 | 0 | 0 | 0 | 1.00 |
| z_RD | clinIC3 | -0.04 | -0.09 | 0.01 | 0.05 | 0.95 | 5.11 | 0 | 0 | 0 | 1.00 |
| z_RD | clinIC4 | 0.05 | -0.00 | 0.09 | 0.97 | 0.03 | 3.10 | 0 | 0 | 0 | 1.00 |
| z_RD | clinIC5 | 0.03 | -0.03 | 0.08 | 0.85 | 0.15 | 10.96 | 0 | 0 | 0 | 1.00 |
| z_RD | clinIC6 | 0.02 | -0.03 | 0.07 | 0.75 | 0.25 | 14.99 | 0 | 0 | 0 | 1.00 |
| z_RD | clinIC7 | 0.05 | -0.01 | 0.09 | 0.96 | 0.04 | 4.40 | 0 | 0 | 0 | 1.00 |
| z_RD | mean_clinICA | 0.03 | -0.03 | 0.09 | 0.89 | 0.11 | 8.72 | 0 | 0 | 0 | 1.00 |
| z_RD | z | 0.09 | 0.03 | 0.15 | 1.00 | 0.00 | 0.15 | 0 | 0 | 0 | 1.00 |
| z_MD | clinIC1 | -0.01 | -0.07 | 0.05 | 0.38 | 0.62 | 16.45 | 0 | 0 | 0 | 1.00 |
| z_MD | clinIC2 | -0.04 | -0.09 | 0.01 | 0.08 | 0.92 | 6.87 | 0 | 0 | 0 | 1.00 |
| z_MD | clinIC3 | -0.07 | -0.12 | -0.02 | 0.00 | 1.00 | 0.62 | 0 | 0 | 0 | 1.00 |
| z_MD | clinIC4 | -0.00 | -0.05 | 0.05 | 0.48 | 0.52 | 19.80 | 0 | 0 | 0 | 1.00 |
| z_MD | clinIC5 | 0.00 | -0.05 | 0.05 | 0.57 | 0.43 | 18.37 | 0 | 0 | 0 | 1.00 |
| z_MD | clinIC6 | 0.02 | -0.04 | 0.07 | 0.73 | 0.27 | 15.24 | 0 | 0 | 0 | 1.00 |
| z_MD | clinIC7 | 0.03 | -0.02 | 0.08 | 0.91 | 0.09 | 8.15 | 0 | 0 | 0 | 1.00 |
| z_MD | mean_clinICA | -0.02 | -0.08 | 0.03 | 0.20 | 0.80 | 11.53 | 0 | 0 | 0 | 1.00 |
| z_MD | z | 0.01 | -0.05 | 0.07 | 0.68 | 0.32 | 14.86 | 0 | 0 | 0 | 1.00 |

**Supplemental table S5**. Summary stats for Bayesian statistical analysis with the deviation scores derived from cortical thickness and surface area as the dependent variable.

| *y* | *clin* | *estimate* | *lower95* | *upper95* | *p_higher_0* | *p_lower_0* | *evidence_0* | *BF_0* | *loo_elpd_diff* | *loo_se_diff* | *max_rhat* |
| --- | --- | --- | --- | --- | --- | --- | --- | --- | --- | --- | --- |
| z_ct | clinIC1 | -0.05 | -0.10 | 0.00 | 0.04 | 0.96 | 3.86 | 0 | 0 | 0 | 1.00 |
| z_ct | clinIC2 | -0.03 | -0.08 | 0.02 | 0.11 | 0.89 | 9.71 | 0 | 0 | 0 | 1.00 |
| z_ct | clinIC3 | -0.03 | -0.08 | 0.02 | 0.13 | 0.87 | 11.27 | 0 | 0 | 0 | 1.00 |
| z_ct | clinIC4 | -0.06 | -0.11 | -0.02 | 0.00 | 1.00 | 0.59 | 0 | 0 | 0 | 1.00 |
| z_ct | clinIC5 | -0.05 | -0.10 | 0.00 | 0.03 | 0.97 | 3.02 | 0 | 0 | 0 | 1.00 |
| z_ct | clinIC6 | -0.03 | -0.07 | 0.02 | 0.15 | 0.85 | 12.16 | 0 | 0 | 0 | 1.00 |
| z_ct | clinIC7 | -0.03 | -0.08 | 0.02 | 0.12 | 0.88 | 10.24 | 0 | 0 | 0 | 1.00 |
| z_ct | mean_clinICA | -0.11 | -0.16 | -0.06 | 0.00 | 1.00 | 0.01 | 0 | 0 | 0 | 1.00 |
| z_ct | z | -0.10 | -0.15 | -0.04 | 0.00 | 1.00 | 0.04 | 0 | 0 | 0 | 1.00 |
| z_sa | clinIC1 | -0.05 | -0.11 | -0.00 | 0.02 | 0.98 | 2.34 | 0 | 0 | 0 | 1.00 |
| z_sa | clinIC2 | -0.10 | -0.15 | -0.05 | 0.00 | 1.00 | 0.00 | 0 | 0 | 0 | 1.00 |
| z_sa | clinIC3 | -0.10 | -0.15 | -0.06 | 0.00 | 1.00 | 0.00 | 0 | 0 | 0 | 1.00 |
| z_sa | clinIC4 | -0.07 | -0.11 | -0.03 | 0.00 | 1.00 | 0.16 | 0 | 0 | 0 | 1.00 |
| z_sa | clinIC5 | 0.01 | -0.04 | 0.06 | 0.71 | 0.29 | 17.17 | 0 | 0 | 0 | 1.00 |
| z_sa | clinIC6 | -0.03 | -0.08 | 0.02 | 0.10 | 0.90 | 8.83 | 0 | 0 | 0 | 1.00 |
| z_sa | clinIC7 | 0.02 | -0.03 | 0.07 | 0.80 | 0.20 | 14.70 | 0 | 0 | 0 | 1.00 |
| z_sa | mean_clinICA | -0.13 | -0.18 | -0.08 | 0.00 | 1.00 | -0.00 | 0 | 0 | 0 | 1.00 |
| z_sa | z | -0.32 | -0.37 | -0.27 | 0.00 | 1.00 | 0.00 | 0 | 0 | 0 | 1.00 |
